# Hand hygiene behavior change: a review and pilot study of an automated hand hygiene reminder system implementation in a public hospital

**DOI:** 10.1017/ash.2023.195

**Published:** 2023-07-10

**Authors:** Arta Seferi, Kalliopi Parginos, Wiline Jean, Christopher Calero, Joshua Fogel, Shantel Modeste, Beverley-Ann Scott, Marjorie Daly-Walsh, Wilfredo Yap, Manjinder Kaur, Terence Brady, Theresa Madaline

**Affiliations:** 1 Department of Nursing, New York City Health + Hospitals/South Brooklyn Health, Brooklyn, NY, USA; 2 Department of Infection Prevention, New York City Health + Hospitals/South Brooklyn Health, Brooklyn, NY, USA; 3 HealthCare Transformation, Chicago, IL, USA; 4 Department of Obstetrics and Gynecology, New York City Health + Hospitals/South Brooklyn Health, Brooklyn, NY, USA; 5 Department of Business Management, Brooklyn College, Brooklyn, NY, USA; 6 Department of Quality Management, New York City Health + Hospitals/South Brooklyn Health, Brooklyn, NY, USA; 7 Department of Medicine, New York City Health + Hospitals/South Brooklyn Health, Brooklyn, NY, USA; 8 Department of Medicine, St. George’s University School of Medicine, Grenada, West Indies; 9 Department of Medicine, Touro College of Osteopathic Medicine, New York, NY, USA; 10 Department of Medicine, New York Institute of Technology College of Osteopathic Medicine, New York, NY, USA

## Abstract

**Objective:**

To review and study implementation of an automated hand hygiene reminder system (AHHRS).

**Design:**

Prospective, nonrandomized, before-after quality improvement pilot study conducted over 6 months.

**Setting:**

Medical-surgical unit (MSU) and medical intensive care unit (MICU) at a public hospital in New York City.

**Participants:**

There were 2,642 healthcare worker observations in the direct observation (DO) period versus 265,505 in the AHHRS period, excluding AHHRS observations collected during the 1-month crossover period when simultaneous DO occurred.

**Intervention:**

We compared hand hygiene adherence (HHA) measured by DO prior to the pilot and after AHHRS implementation. We compared changes in HHA and potential cross-contamination events (CCEs) (room exit and subsequent entry without HHA) from baseline for each biweekly period during the pilot.

**Results:**

Engagement, education/training, data transparency, and optimization period resulted in successful implementation and adoption of the AHHRS. Observations were greater utilizing AHHRS than DO (265,505 vs 2,642, *P* < .01). Due to the expected Hawthorne effect, HHA was significantly less for AHHRS than DO in MSU (90.99% vs 97.21%, *P* < .01) and MICU (91.21% vs 98.65%, *P* < .01). HHA significantly improved from 86.47% to 89.68% in MSU (*P* < .001) and 85.93% to 91.24% in the MICU (*P* < .001) from the first biweekly period of AHHRS utilization to the last. CCE decreased from 73.42% to 65.11% in the MSU and significantly decreased from 81.22% to 53.19% in the MICU (*P* < .05).

**Conclusions:**

We describe how an AHHRS approach was successfully implemented at our facility. With ongoing feedback and system optimization, AHHRS improved HHA and reduced CCE over time.

## Introduction

The Centers for Disease Control and Prevention (CDC) estimates 1 in 31 hospitalized patients acquire a hospital-acquired infection (HAI) every year in the United States.^1^ These preventable infections result in 99,000 deaths^2^ and $30 billion spent yearly.^3^ Transmission of pathogens from the hands of healthcare workers (HCWs) to patients, and prevention of transmission through handwashing, was established in the 1800s by Ignaz Semmelweis.^4^ Consistent performance of hand hygiene (HH) by HCWs remains a major barrier to reducing HAIs today.^5^ HCWs can overlook this crucial patient safety measure due to high patient care demands, long hours, and structural barriers.^6^ A review reports that HH adherence (HHA) among HCWs is approximately 50%.^7^ Furthermore, HCWs are also at risk of self-contamination or infection. HH can prevent an exposure or infection in the clinical setting from gram-negative bacilli, methicillin-resistant *Staphylococcus aureus* (MRSA), vancomycin-resistant enterococci (VRE), and *C. difficile*.^8–13^ HH can protect HCW from acquisition of influenza, respiratory syncytial virus (RSV), severe acute respiratory syndrome (SARS), and COVID-19 during patient care.^14,15^

## Hand hygiene measurement approaches

### Direct observation (DO)

HH programs are a well-established infection prevention practice recommended by many organizations including the World Health Organization (WHO), the CDC, Infectious Diseases Society of America, and the American Hospital Association.^4,16–18^ Direct observation (DO) of HH among HCWs is the gold standard for HH programs. This strategy has several advantages: (1) opportunity for real-time feedback and education, (2) observation of all five of WHO’s moments for HH (before patient contact, before aseptic task, after body fluid exposure risk, after patient contact, and after contact with patient surroundings), (3) evaluation of technique and product used, and (4) assess for appropriate use of gloves and other personal protective equipment.^4,16,18–21^ However, DO for HH performance is limited by the Hawthorne effect,^18,22–24^ which is that the person under observation changes behavior due to presence of the observer. As HHA rates are higher when HCWs are aware they are being observed than when they are unaware,^24^ the practical utility of DO programs has limitations. Furthermore, DO is costly, labor-intensive, potentially subjective, and misses a significant number of opportunities, and the limited availability of observers can result in observations being performed disproportionately on certain days/times or locations.^18,19^

### Technology-assisted monitoring

Technology-assisted HHA monitoring strategies are used to overcome some barriers of DO, including reduction in the Hawthorne effect, decrease in the time of data collection, and increase in the opportunities captured.^18,25–28^ However, these strategies require financial resources,^18,19,25–28^ and the use of remote monitoring, product usage, or electronic counting devices alone without feedback resulted in low HHA rates.^29–32^ Only when individual feedback was provided did HH rates increase.^28^ Several technology systems are available to both provide real-time automated reminders to HCW to perform HH and collect adherence data remotely that is shared with the end-user.^30,33–36^ These automated hand hygiene reminder systems (AHHRSs) typically include wearable devices that provide a vibration, light, and/or audible sound to the user as a reminder to perform HH,^33–36^ and some include alcohol sensors for detection of alcohol-based hand rub.^35^ Feasibility studies of AHHRS indicate low error rates and high rates of HH performance, in addition to the large numbers of HHO captured as compared to DO.^37–40^ HCW feedback for AHHRS optimal design and implementation include: (1) wearing a small and unobtrusive device for HH would demonstrate professional accountability, (2) the device should offer a vibratory or audible reminder to perform HH, (3) the device should capture opportunities around the patient’s physical location, (4) there should be a period of time to become accustomed to the device before performance measurement begins, (5) confidential audit and feedback of the user’s own performance should be provided, and (6) need for transparency regarding who would have access to adherence data and what the data would be used for in the form of clear policies and procedures prior to implementation.^41,42^ Previous reports of AHHRS implementation with end-user feedback as compared to DO demonstrate greater number of observations captured,^40,43^ lower adherence measurement due to the Hawthorne effect,^40^ reduction in HAI,^43–45^ and reduction in HCW sick days.^46^ However, there are no studies examining the unique impact of AHHRS-issued reminders on cross-contamination events (CCEs) over time.

We aim to assess the impact of a collaborative implementation strategy of AHHRS on overall HHA performance and potential CCEs in both a medical-surgical unit (MSU) and a medical intensive care unit (MICU). We hypothesize that engagement, education/training, data transparency, and optimization period will result in successful implementation and adoption of an AHHRS, HHA measurement with AHHRS will be lower than DO (due to the Hawthorne effect), HHA with the use of AHHRS will improve after baseline, and CCE will decrease over time with end-user feedback and real-time alerts.

## Methods

### Setting

This was a prospective, nonrandomized, before-after quality improvement feasibility pilot project conducted at NYC Health + Hospitals South Brooklyn Health, a 371-bed public hospital in New York City. Two units were identified for participation in the pilot: MSU and the MICU. These locations were chosen based on high rates of HAI in these areas of the hospital in the preceding year. As this was a quality improvement project, Institutional Review Board approval was not required.

### Intervention and selection of AHHRS

A multidisciplinary team of nurses, physicians, infection-prevention specialists, educators, information-technology specialists, and environmental services associates (EVSs) assessed and selected an AHHRS platform. From September to December 2020, the team performed a risk/benefit analysis of existing AHHRS platforms, utilizing end-user feedback and feasibility, existing HH research studies, HAI outcome data, occupational safety data, data management process and security, opportunities for optimization and adjustment, costs, and expert opinion^30,32,40,42–46^ to select the BioVigil AHHRS (BioVigil Technologies, Ann Arbor, MI) (Table 1).

**Table 1.** Risk/benefit analysis of BioVigil's automated hand hygiene reminder system

Benefit	Risk
Able to capture most HH opportunities^29,30^	Financial investment for equipment and data management^32^
Provides vibratory, audible, and visual reminders to correct a missed opportunity and correct behavior^30,35,40^	Requires education and training to be effective^45^
Configurable to the hospital’s HH policy	Reliant on wearer to use
Adjustable settings based on staff feedback	No human-to-human education opportunity during observation
Can distinguish and measure if the appropriate type of HH was performed based on patient isolation status (eg, soap and water vs alcohol-based hand rub)	Staff concerns regarding privacy and data transparency^32^
Captures wearer’s role and utilization	Requires upfront information on employee roles and ongoing maintenance of employee lists
Provides aggregated feedback on performance to end-users	Limited ability to measure the quality and effectiveness of HH technique beyond sufficient alcohol-based hand rub quantity and duration of soap and water hand washing.
Can configure security and access to limited personnel (infection prevention, senior leadership) consistent with institutional policies and procedures	Cannot assess adherence to glove or nail policies
Can be used to investigate outbreaks or HAIs when necessary	Will miss some moments for HH opportunities occurring in the same episode of care (eg, after performing tasks with high risk of contamination, before an aseptic task, after touching the environment, etc)
Does not interfere with end-user workflow	Space, power, and data connection required on each unit/area for base stations
Meets system security requirements	
Minimal equipment installation requirements and environmental risks	
Front facing to include patients and loved ones	
Study data supports HAI reduction (*C. difficile*, CAUTI, and CLABSI)^43,44^	
Study data supports prevention of employee illness^46^	

Note. HH, hand hygiene; HAI, hospital-acquired infection; CAUTI, catheter-associated urinary tract infection; CLABSI, central line-associated bloodstream infection.

**Figure 1.**
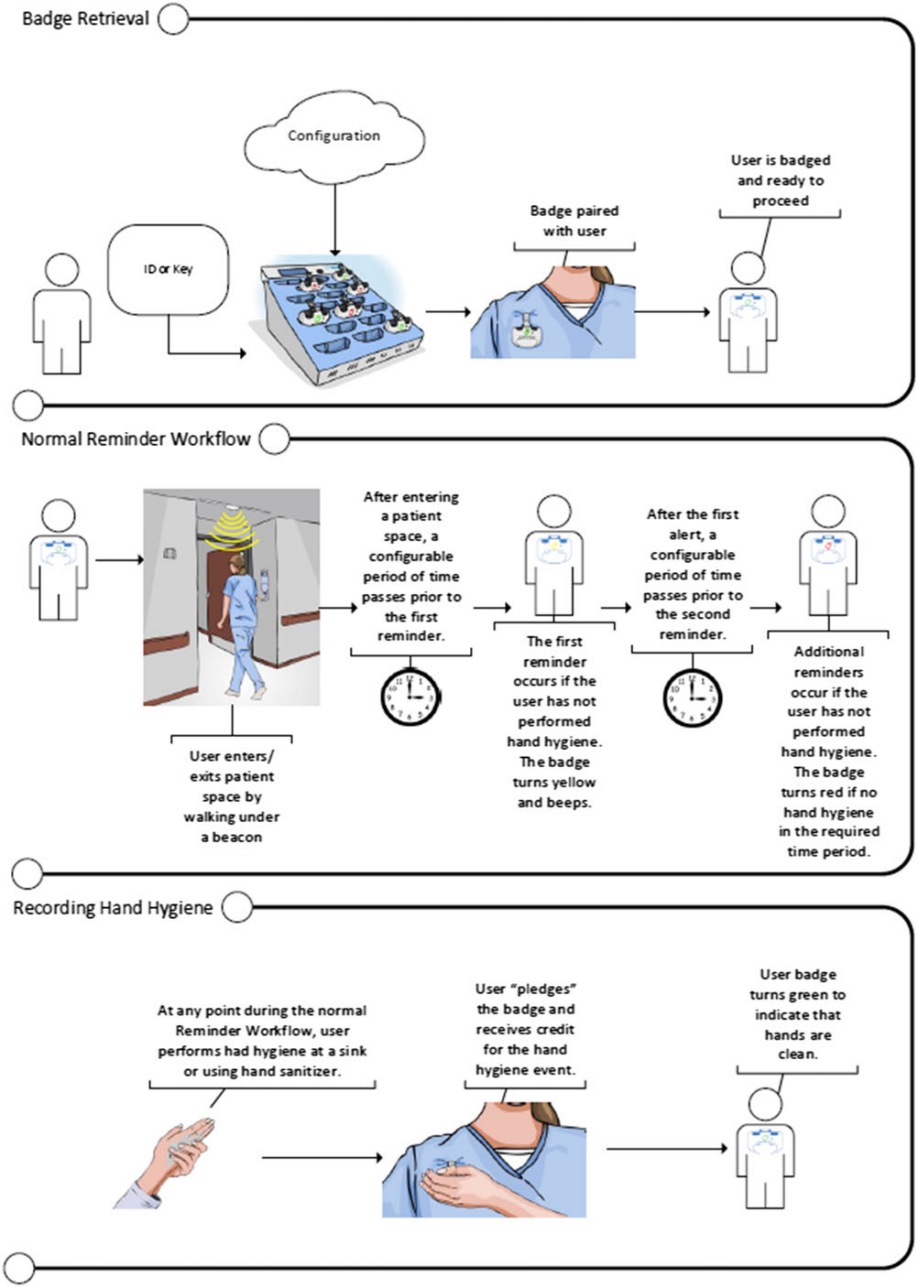
BioVigil: how it works diagram. Copyright © BioVigil LLC; Reproduced with permission.

BioVigil is an AHHRS that utilizes a wearable badge that provides vibration, audible, and visual reminders if HH is not detected within a specified time after entering or exiting a patient room or moving between patient zones. The badge communicates with beacons in doorways, at the head of patient beds, and with handwashing sinks when soap and water HH is required. The badge determines adequacy of HH based on sufficient alcohol concentration when using sanitizer, as well as duration spent at the handwashing sink when using soap and water. Data are collected in real time and transmitted virtually once the badge is placed back in a base station for charging (Figure 1). The badges are configurable to an institution’s HH policy, user feedback, role, location, and isolation status and include an exception function for emergencies or when HH is not required (eg, entering empty rooms). Configuration is performed at the time of installation, and isolation status is easily updated using a remote device that interfaces with the room beacon. Badges do not have location tracking capability or transmit information other than HH performance data. End-users receive feedback on their HH performance and badge utilization via email; other users can receive aggregated data as configured and determined by the institution’s policies. Color-changing badge indicators of adherence status empower patients in HHA.

The team developed an implementation plan based on the existing literature.^20,21,30,42,47^ The strategy included staff engagement ahead of AHHRS installation (March–July 2021), formal education and training (July 21–23, 2021), transparency on how and when data from the pilot would be shared with leaders and end-users (July 21–23, 2021), and a training period whereby users could practice using the device in advance of AHHRS data collection and provide recommendations for optimization of settings prior to formal go-live (July 21–July 31, 2021). A crossover period of 1 month during which both AHHRS and DO were utilized for HHA was included to ensure adequate HH monitoring occurred in the event of technical or other difficulties impacting data collection (August 1–31, 2021). After go-live (August 1, 2021), pilot participants received adherence status, CCE, and device utilization hours data biweekly by email. Leaders received weekly HHA reports, daily CCE reports, and monthly leadership overviews.

### Pre-implementation engagement

Biweekly town hall meetings, daily unit-based huddles, biweekly leadership rounds, and two labor union meetings were conducted prior to the installation and training for the AHHRS beginning in March 2021. Staff were given written tip sheets about the AHHRS, and patients were given written information in admission packets about the pilot and how the AHHRS works. Data were shared on HAIs, and the difference between rates of HHA when staff was aware and unaware of observations. Information about the AHHRS was shared with all stakeholders as well as patients and caregivers, including the reasons for the pilot: protection of both staff and patients in the setting of COVID-19 and high HAI rates, and to ensure the system was acceptable to end-users and effective before adoption institution-wide.

### Education and training

The AHHRS equipment was installed on July 19, 2021, and the system was configured to match the institution’s HH policy. The initial settings in the AHHRS allowed 90 seconds following entry to perform HH, 60 seconds grace period after room entry to exit a room without performing HH for the purposes of rounding without patient or environmental contact, and a minimum of 20 seconds at sinks for soap and water HH. Education took place through online module followed by in-person training. The online module consisted of a video describing the AHHRS purpose, functionality, and end-user workflow as well as a 13-question knowledge assessment with an agreed-upon passing score of 12 correct.

### Data transparency

During training sessions, huddles, and rounds, staff was informed about exactly which data elements would be collected by the AHHRS, how often performance reports would be issued by email, how feedback reports would appear, and who would receive adherence data other than each individual end-user. Staff were assured that no punitive actions would be taken based on the pilot and the data collected by the AHHRS. Leaders subsequently utilized summary reports during the AHHRS pilot period to provide high-level feedback about unit performance, celebrate and award high-performers, and encourage staff to provide feedback about the AHHRS during daily huddles.

### Optimization

Pilot participants began wearing and practicing use of the AHHRS badges from July 24 to July 31, 2021, prior to formal initiation of data collection. End-user recommendations to improve accuracy and usability were implemented prior to go-live.

### Participants

HCWs in roles with the greatest patient contact and/or highest risk for contamination were included in the pilot: registered nurses (RNs), licensed practical nurses (LPNs), certified-nurse aides (CNAs), personal-care assistants (PCAs), physicians including trainees (MDs), physician assistants (PAs), respiratory therapists (RTs), and EVSs.

### Variables and outcome variables

Staff member participation by role was quantified for the DO and AHHRS periods. During AHHRS implementation, educational opportunities, competency assessments, and changes to the AHHRS system based on staff feedback were described. HHO (patient room entry or room exit) and HHA (performance of appropriate HH upon room entry or exit) of HCW were quantified for the DO control (March–June 2021) and AHHRS intervention (September–December 2021) periods. Opportunities and adherence captured during the 1 month (August 2021) of crossover where both DO and AHHRS occurred were omitted from the comparative analysis of DO versus AHHRS measurement to allow for measuring the impact of each measure alone.

HHA during each biweekly period of the AHHRS pilot (August–December 2021) was measured as well as potential cross-contamination opportunities (CCOs) and CCE, and time to HHA after room exit. CCOs were defined as exit from one patient room without HHA followed by subsequent entry to another patient room; CCEs were defined as a CCO without HHA after entry into the second patient room.

### Statistical analysis

Categorical variables were described as frequencies and percentages. The Pearson χ^2^ statistic compared the DO approach with the AHHRS approach for HHO participant categories and HHA. The McNemar test compared the baseline biweekly percentage to each of the additional 10 biweekly percentages for the AHHRS approach. The summary data were analyzed with immediate commands of tabi and mcci. Stata/SE Version 17 was used for the analyses (StataCorp, College Station, TX, 2021).

## Results

### Education and training

Over 92% (284/307) of participating staff members completed online training and passed the knowledge test. There were 5 live education sessions conducted daily from July 21 to 23, 2021, covering staff on all 3 shifts for 15 total sessions. The live sessions included how to endorse the device to capture HH events, time frames for the presence of alerts, when nonadherence is considered, workflow allowances such as emergencies, practice using the device, and teach-back demonstration. There were 75.2% (231/307) participating staff members who attended a live session, and the remainder were trained individually on the unit in the following week.

## Optimization

Four leadership rounds and 16 huddles were conducted during the period between training and formal go-live where end-users were encouraged to provide feedback and recommendations. While users did not report interference with daily workflow, recommendations included adjusting settings to allow sufficient time for PPE donning, improving accuracy of beacon activation at handwashing sinks, and more frequent monitoring and refilling of hand sanitizer and soap dispensers by EVSs. The AHHRS settings and equipment were optimized based on staff feedback including extending entry time from 90 seconds to 120 seconds, changing the location of beacons above sinks for improved detection, and twice daily rounding of EVSs to refill hand sanitizer and soap dispensers. These changes were communicated with participating staff, empowering them as partners in the success of the pilot. Validation of DO and AHHRS confirmed that the recommended changes resulted in accurate capture of HHA.

### Data collection and feedback

#### Distribution of HHO by Role

The distribution of HHOs and participation in the pilot program across HCW roles is provided in Table 2. The significance for observations comparisons (*P* < .001) showed that DO had a greater percentage for MD/PA and that AHHRS had a greater percentage for RN/LPN. There were 2,642 observations in the DO period versus 265,505 HHOs collected by the AHHRS; these AHHRS observations excluded any AHHRS observations collected during the crossover period in August 2021 where simultaneous DOs occurred.

**Table 2.** HH observation comparison and pilot participation by healthcare worker role

Healthcare worker role	Direct observations frequency (percentage)	Automated observations frequency (percentage)	Pilot participants for automated observations frequency (percentage)
Registered nurses and licensed practical nurses	957 (36.22)	183,446 (69.09)	72 (23.45)
Certified nurse aides and personal care assistants	279 (10.56)	46,231 (17.41)	25 (8.14)
Physicians (including trainees) and physician assistants	671 (25.40)	5,197 (1.96)	145 (47.23)
Respiratory therapists	68 (2.57)	10,396 (3.92)	38 (12.38)
Environmental services associates	73 (2.76)	3,073 (1.16)	11 (3.58)
Other disciplines or unassigned	594 (22.48)	17,162 (6.46)	16 (5.21)

Note. Direct observation categories significantly differed from automated observation categories (*P* < .001).

#### Comparison of HHA between monitoring systems

Pearson χ^2^ comparisons for HHA between the DO and the AHHRS approaches where exclusively one observation approach was used are shown in Table 3. Overall HHA for DO (97.65%) was significantly greater compared to AHHRS (91.04%) (*P* < .001). In the MICU and the MSU, HHA for DO was significantly greater compared to AHHRS (98.65% vs 91.21%, *P* < .001 and 97.21% vs 90.99%, *P* < .001).

**Table 3.** HHA comparisons between direct observation and the AHHRS

Variable	Direct frequency (percentage)	Automated frequency (percentage)	*P* value
Whole sample			<.001
* *No	62 (2.35)	23,777 (8.96)	
* *Yes	2,580 (97.65)	241,728 (91.04)	
Medical ICU			<.001
* *No	11 (1.35)	5,777 (8.79)	
* *Yes	805 (98.65)	59,925 (91.21)	
Medical-surgical unit			<.001
* *No	51 (2.79)	18,000 (9.01)	
* *Yes	1,775 (97.21)	181,803 (90.99)	

Note. ICU, intensive care unit. Pearson χ^2^ analyses used for *P* value.

#### Change in HHO and HHA over time

**Figure 2.**
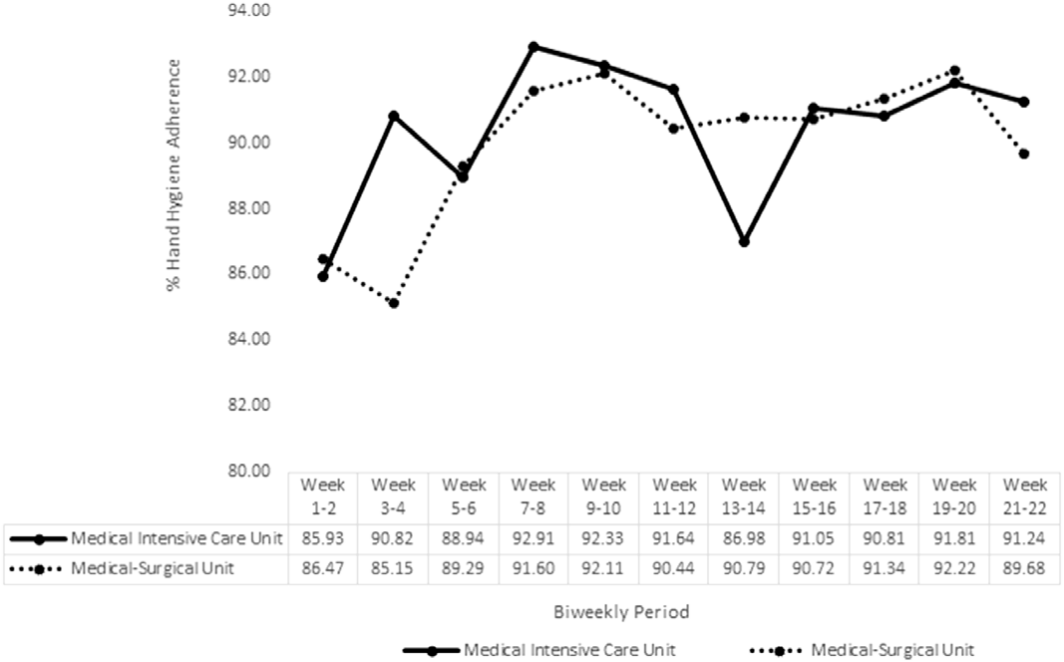
Percent HHA for the AHHRS.

Overall volume of the AHHRS HHO captured decreased over time from the first biweekly period to the last full biweekly period, which was the second-to-last biweekly period of weeks 19–20 (MICU: 12,315–4,678; MSU: 32,058–19,616). HHA biweekly percentages for the AHHRS period beginning from go-live in August are shown in Figure 2. For MICU, biweekly HHA was lowest during baseline weeks 1–2 (85.93%), highest during weeks 7–8 (92.91%), and a 5.31% greater percentage point during the last biweekly period (91.24%) than baseline. McNemar test comparisons from baseline to each additional 10 biweekly periods were all statistically significant (all *P* < .001); all 10 biweekly periods showed HHA significantly increased from baseline. For MSU, biweekly HHA was lowest during weeks 3–4 (85.15%), highest during weeks 19–20 (92.22%), and a 3.21% greater percentage point during the last biweekly period (89.68%) than baseline. McNemar test comparisons from baseline to each additional 10 biweekly percentages were all statistically significant (all *P* < .001). One biweekly period showed HHA significantly decreased from baseline, while 9 biweekly periods showed HHA significantly increased from baseline.

#### Change in CCE over time

**Figure 3.**
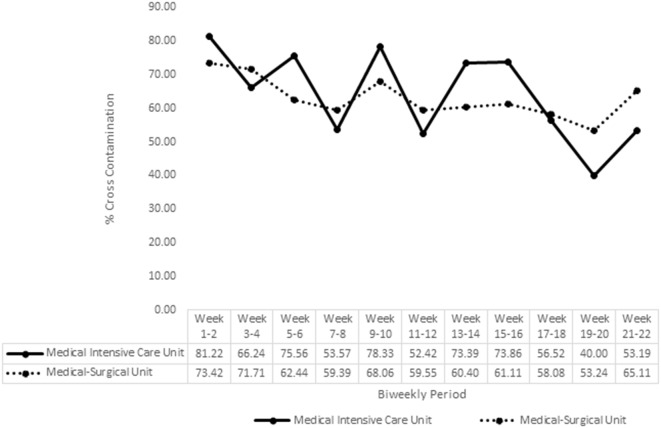
Percent cross-contamination for the AHHRS.

Figure 3 shows CCE for the AHHRS period beginning from go-live in August. For MICU, CCE during baseline weeks 1–2 was 81.22%. Using the McNemar test and compared from baseline, CCE significantly decreased (either *P* < .001, *P* < .01, or *P* < .05) for 8 biweekly periods. However, CCE for biweekly periods of weeks 11–12 and weeks 15–16 did not significantly differ from baseline. The last biweekly period of weeks 21–22 had CCE of 53.19% for a percentage point decrease of 28.03% from baseline. Although all biweekly time periods had decreased CCE from baseline, there were biweekly time periods where a decrease was followed by an increase in the next biweekly time period. For MSU, CCE during baseline weeks 1–2 was 73.42%. Using the McNemar test and compared from baseline, CCE significantly decreased for 7 biweekly periods (either *P* < .001 or *P* < .01). However, CCE for biweekly periods of weeks 13–14, weeks 17–18, and weeks 21–22 did not significantly differ from baseline. The last biweekly period of weeks 21–22 had a CCE of 65.11% for a percentage point decrease of 8.31% from baseline.

#### Time to HHA analysis

**Figure 4.**
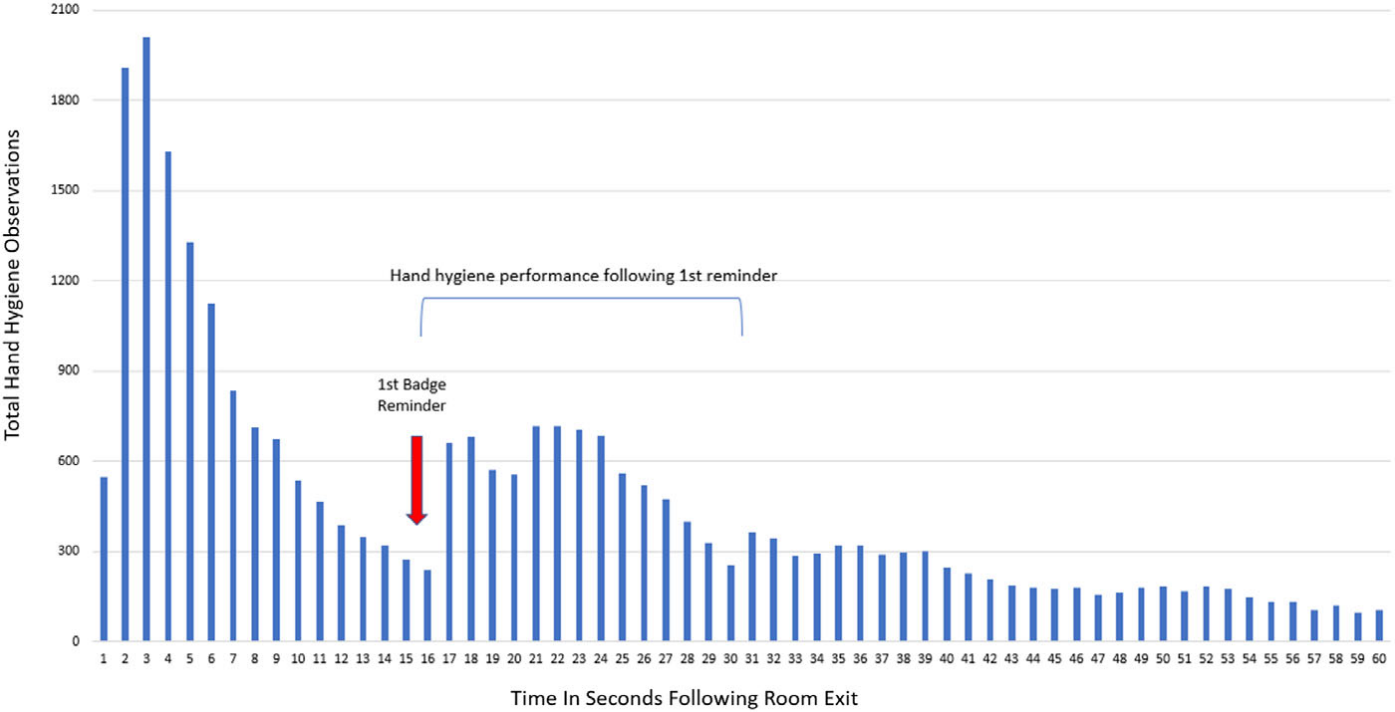
Time to HHA analysis for all room exit observations.

Time to HHA analysis for all room exit observations during the pilot period indicated that HH was most commonly performed within 9 seconds of exit, but a reminder after 15 seconds produced another spike in HH events in the first 24 seconds after exit, suggesting that the reminder produces a change in HH behavior (Figure 4). There were 34.70% (3,147/9,068) of potential CCE that occurred during the AHHRS period that were corrected after a badge reminder, potentially preventing pathogen transmission.

## Discussion

We found support for our hypothesis that engagement, education/training, data transparency, and optimization period resulted in successful implementation and adoption of the AHHRS. Following successful implementation, AHHRS HHA improved from baseline, consistent with our hypothesis. Our observed HHA percentages of approximately 90% are similar to previous AHHRS studies, supporting the concept that automated reminders and feedback are effective in achieving high rates of HHA in different clinical settings.^35,40,44^ We suggest that consistent real-time end-user feedback during patient care and reminders when HH is inadvertently missed result in high rates of HHA while using the AHHRS, as supported by the bimodal time-to-HHA analysis in our study. As hypothesized, we observed HHA measurement with AHHRS was lower than DO, this is most likely due to the known impact of the Hawthorne effect on DO-based measurements.

This study is the first to evaluate CCE using an AHHRS and to demonstrate an improvement in CCE over time. Reduction in CCE could be an underlying cause of the reduced HAI observed in other AHHRS studies, and further prospective study of this association is warranted. There was greater variation in biweekly CCE in the MICU than in the MSU. One potential reason is higher acuity of patients requiring rescue and failure to utilize the exception function on AHHRS badges; CCE variation in different clinical settings is an area for future investigation in the future.

We observed that RN/LPNs (69.09%) and CNA/PCAs (17.01%) had the most frequent AHHRS events, and MD/PAs (1.96%) and EVSs (1.06%) had the least frequent AHHRS events. We suggest that differences in frequency of room entry by role and exclusion of MD/PA and EVS staff who were not assigned to the unit consistently are responsible for these observed differences. Literature on DO showed similar differences in HHO by role^6^; this could be an area for further investigation using AHHRS.

Cost of AHHRS and other technology-assisted HH monitoring strategies is a limitation to widespread adoption and implementation.^18,32^ However, DO programs have high costs too. At our institution, to meet Leapfrog (an advocacy group for healthcare safety), HHO requirements (200 observations per unit monthly)^48^, at an estimated 10 minutes per event and 16 clinical units, require an estimated 533 hours of employee time monthly.^49^ Data entry and analysis, report generation, and data distribution are another estimated 5 hours per unit monthly, adding an additional 80 hours of employee time monthly. This is approximately 613 hours of employee time monthly for DO. The cost (based on $37.50/h average hourly compensation rate for nurses performing these observations)^50^ is $22,987.50 monthly, or $275,850 yearly for our DO program. Furthermore, multimodal HH strategies using AHHRS improve HHA rates and reduce HAI.^43–45^ Cost savings from HAI reductions should be included in financial analysis of AHHRS. Prior studies of AHHRS demonstrate a 45% reduction in central line-associated bloodstream infection,^44^ 55% reduction in catheter-associated urinary tract infection,^44^ and 38% reduction in *Clostridioides difficile* infection.^43^

Our quality improvement project had several limitations and practical challenges. First, the intervention was not designed to measure changes in clinical outcomes, HAI, or employee illness. Second, there was a decrease in AHHRS utilization over time. It is likely that staff turnover, increase in agency staff during COVID-19 surges, and rotation of nurses and housestaff to different units or hospitals impacted utilization; future studies should formally evaluate utilization over time by measuring HCW-specific events. Third, a randomized prospective study design with a longer trial period, more data points, and more disciplines included would provide greater clarity and depth of knowledge regarding changes in behavior with AHHRS. Fourth, double-bedded rooms posed challenges in accurate capturing of HHO, despite changes in position of beacons to reduce inconsistencies. Lastly, our analysis did not include potential employee illness reduction cost savings or avoidance of Centers for Medicare and Medicaid Services penalties through the hospital-acquired condition reduction program or related programs, likely underestimating true return-on-investment for AHHRS implementation.

In conclusion, an AHHRS approach was successfully implemented at our healthcare facility. With ongoing feedback and system optimization, AHHRS improved HHA and reduced CCE over time.
